# The rs2442598 polymorphism in the *ANGPT-2* gene is associated with risk for diabetic retinopathy in patients with type 1 diabetes mellitus in a Brazilian population

**DOI:** 10.20945/2359-3997000000417

**Published:** 2021-11-11

**Authors:** Cristine Dieter, Natália Emerim Lemos, Nathalia Rodrigues de Faria Corrêa, Aline Rodrigues Costa, Luís Henrique Canani, Daisy Crispim, Andrea Carla Bauer

**Affiliations:** 1 Hospital de Clínicas de Porto Alegre Divisão de Endocrinologia Porto Alegre RS Brasil Divisão de Endocrinologia, Hospital de Clínicas de Porto Alegre, Porto Alegre, RS, Brasil; 2 Universidade Federal do Rio Grande do Sul Faculdade de Medicina Programa de Pós-graduação em Ciências Médicas – Endocrinologia Porto Alegre RS Brasil Programa de Pós-graduação em Ciências Médicas – Endocrinologia, Faculdade de Medicina, Universidade Federal do Rio Grande do Sul, Porto Alegre, RS, Brasil; 3 Hospital de Clínicas de Porto Alegre Divisão de Nefrologia Porto Alegre RS Brasil Divisão de Nefrologia, Hospital de Clínicas de Porto Alegre, Porto Alegre, RS, Brasil

**Keywords:** *ANGPT-2* gene, polymorphism, type 1 diabetes mellitus, diabetic retinopathy

## Abstract

**Objective:**

As studies have reported the involvement of angiopoietin-2 (ANGPT-2) in the pathogenesis of diabetic retinopathy (DR), the aim of this study was to investigate the association between the *ANGPT-2* rs2442598 polymorphism and DR.

**Materials and methods:**

This case-control study comprised 107 patients with type 1 diabetes mellitus (T1DM) and DR (cases) and 129 patients with T1DM without DR (controls) and with ≥ 10 years of DM. The *ANGPT-2* rs2442598 (G/A) polymorphism was genotyped by real-time PCR using TaqMan MGB probes.

**Results:**

Genotype distributions of this polymorphism were consistent with the Hardy-Weinberg equilibrium. The frequency of the rs2442598 A allele was higher in cases compared to controls (p = 0.011). Moreover, the A/A genotype was more frequent in cases than in controls (p = 0.017) and was associated with risk for DR after adjustments for duration of DM, HbA1c, triglycerides, estimated glomerular filtration rate, and hypertension (odds ratio [OR] = 5.19, 95% confidence interval [CI] 1.21-22.27). This association was maintained under recessive (OR = 4.78, 95% CI 1.14-19.99) and additive (OR = 6.861, 95% CI 1.45-32.38) inheritance models.

**Conclusion:**

Our data demonstrated, for the first time, an association between the *ANGPT-2* rs2442598 A allele and risk for DR in T1DM patients from southern Brazil. Additional studies are necessary to replicate this association in other populations.

## INTRODUCTION

Diabetic retinopathy (DR) is one of the most disabling microvascular complications of diabetes mellitus (DM) (1). According to the International Diabetes Federation, DR is the leading cause of blindness in working-age adults (2). Clinically, DR is characterized by the presence of typical retinal microvascular signs, such as microaneurysms, hemorrhages, cotton-wool spots, hard exudates, and neovascularization (1). The development and progression of DR depend on the complex interaction of clinical risk factors, environmental factors, and genetic factors (1,3). In this context, the identification of new genetic polymorphisms associated with DR can contribute to a better understanding of the risk factors and predisposition to this diabetic complication.

Of particular interest for DR are genes related to angiogenesis and vascular development. Among them, angiopoietins (ANGPT) are a family of glycoproteins mainly expressed in endothelial cells, which play an important role in angiogenesis and in the regulation of vascular remodeling by binding to the endothelial receptor tyrosine kinase-2 (Tie-2) (4,5). Tie-2 is highly expressed in endothelial cells and demonstrates strong kinase activity. It becomes phosphorylated on several cytoplasmic tyrosine residues, resulting in the activation of pathways related to the inhibition of *de novo* blood vessel growth and vascular hyperpermeability such as PI3/AKT and ERK (5,6). ANGPT-1 is involved in vascular maturation, promotes endothelial cell (EC) survival and migration, inhibits EC apoptosis, limits vascular permeability, and exerts anti-inflammatory effects on EC [review in (5,7)]. In contrast, ANGPT-2 can antagonize the effect of ANGPT-1 on Tie-2 receptors and may destabilize the vasculature [review in (5,7)]. Evidence suggests that ANGPT-2, in conjunction with vascular endothelial growth factor (VEGF), can induce vascular sprouting, while ANGPT-2 in the absence of VEGF is associated with EC apoptosis (7,8).

A number of studies have reported the involvement of *ANGPT-2* in DR (5,7,9,10). Hammes and cols. (10) demonstrated ANGPT-2 to be upregulated in the retina of diabetic rats compared to controls. Rangasamy and cols. (9) also showed that *ANGPT-2* was increased in the retina of diabetic rats after 8 weeks of DM and in human retinal endothelial cells cultured under high glucose conditions. Single nucleotide polymorphisms (SNPs) in the *ANGPT-2* gene may thus be associated with DR susceptibility. rs2442598 is an intron SNP and one of the most studied variants of the *ANGPT-2* gene in different diseases, being associated with risk for rheumatoid arthritis (11), psoriasis vulgaris (12), and acute lung injury (13). However, to date, no study has analyzed SNPs in the *ANGPT-2* gene in patients with DR. Therefore, we investigated for the first time the association between the rs2442598 SNP in *ANGPT-2* and DR in patients with type 1 DM (T1DM).

## MATERIALS AND METHODS

### Participants and clinical and laboratory analyses

This case-control study was performed following STROBE and STREGA guidelines for association studies (14,15). A total of 236 participants, including 107 patients with T1DM and DR (cases) and 129 patients with T1DM but no DR and with more than 10 years of DM diagnosis, were enrolled in this study. All patients were recruited at *Hospital de Clínicas de Porto Alegre* (Rio Grande do Sul, Brazil) between January 2005 and December 2013.

T1DM was diagnosed according to American Diabetes Association (ADA) guidelines (16). The diagnosis of DR was made by an experienced ophthalmologist using fundoscopy (eye fundus examination) through dilated pupils. DR was classified as “absent DR” (without fundus abnormalities), “nonproliferative DR” (NPDR) (microaneurysms, hemorrhage, and hard exudates), or “proliferative DR” (PDR) (newly formed blood vessels and/or growth of fibrous tissue into the vitreous cavity). DR classification was based on the most severely affected eye, according to the Global Diabetic Retinopathy Group scale (17). The Research Ethics Committee at Hospital de Clínicas de Porto Alegre approved the study protocol, and all participants provided assent and written informed consent prior to inclusion in the study.

A standard questionnaire was applied to collect information regarding age, age at T1DM diagnosis, and drug treatments. All patients were subjected to physical examination and laboratory tests, as described previously (18). Serum and plasma samples were collected after 12 h fasting for laboratory analyses (18). Glucose levels were measured using the glucose oxidase method. Glycated hemoglobin (HbA1c) measurements were performed by different methods and the results were traceable to the Diabetes Control and Complications Trial (DCCT) method by offline calibration or through conversion formulae (19). Creatinine was measured by the Jaffé reaction; total plasma cholesterol, high-density lipoprotein (HDL) cholesterol, and triglycerides by enzymatic methods, and urinary albumin excretion (UAE) by immunoturbidimetry (Sera-Pak immuno microalbuminuria, Bayer, Tarrytown, NY, USA; mean intra- and inter-assay coefficients of variance of 4.5% and 11%, respectively) (20). The estimated glomerular filtration rate (eGFR) was calculated using the Chronic Kidney Disease Epidemiology Collaboration (CKD-EPI) equation (21).

### Genotyping

Total DNA was extracted from peripheral blood leukocytes using a standardized salting-out procedure (22). The *ANGPT-2* rs2442598 SNP (G/A) was genotyped using TaqMan SNP Genotyping Assay 20x (Assay ID = C__15803341_10, Thermo Fisher Scientific, Foster City, CA, USA). Real-time polymerase chain reactions (PCRs) were performed in 384-well plates, in a 5 µL volume, using 2 ng of DNA, the TaqPath ProAmp Master Mix (Thermo Fisher Scientific) 1x, and the TaqMan SNP Genotyping Assay 1x (Thermo Fisher Scientific). Plates were then placed in a real-time PCR thermal cycler (ViiA7 Real-Time PCR System; Thermo Fisher Scientific) and heated for 10 min at 95 °C, followed by 50 cycles of 95 °C for 15 s and 62 °C for 1 min.

### Statistical analysis

Allele frequencies were calculated by gene counting, and departures from the Hardy-Weinberg equilibrium (HWE) were verified using the χ^2^ test. Allele and genotype frequencies were compared between groups using χ^2^ tests. Additionally, genotypes were compared between case and control groups under additive, recessive, and dominant inheritance models, categorized as suggested by a previous publication (23). Clinical and laboratory characteristics were compared between groups of patients categorized according to the different SNP genotypes using an unpaired Student’s *t*-test, one-way analysis of variance (ANOVA), or the χ^2^ test, as appropriate. Variables with normal distribution are shown as means ± standard deviations (SDs) or percentages. Variables with skewed distribution were log-transformed before analyses and are shown as medians (25th-75th percentile values). Multivariate logistic regression analyses were done to evaluate the independent association of the rs2442598 SNP with DR, adjusting for possible confounding factors. Variables with significant associations with DR in the univariate analysis or with relevant biological association with this complication were chosen for inclusion in the multivariate model. Statistical analyses were performed using SPSS 18.0 software (SPSS, Chicago, IL, USA), and p-values < 0.05 were considered significant.

Sample sizes were calculated using the OpenEpi website (www.openepi.com). Since no studies have yet investigated the association between the rs2442598 SNP and DR, we used frequencies published by previous studies that assessed the association of this SNP with other diseases (mean minor allele frequency = 0.30 and odds ratio [OR] = 2.2) (11,13). Therefore, the calculated sample size was estimated as 108 participants per group.

## RESULTS

### Sample description

The clinical and laboratory characteristics of patients with T1DM with and without DR are shown in Table 1. Men comprised 52.3% of the cases and 53.5% of the control group (p = 0.964). The mean age of participants was higher in cases with DR when compared to controls (41.4 ± 12.1 vs. 34.6 ± 12.2 years, p < 0.001). As expected, HbA1c, T1DM duration, triglycerides, total cholesterol, low-density lipoprotein (LDL) cholesterol, and UAE values were higher in cases with DR when compared to controls (all p-values < 0.038). The prevalence of hypertension was also higher in patients with DR (p = 0.005), and the eGFR was lower in patients with T1DM and DR when compared to those without DR (p < 0.0001).

**Table 1:** Clinical and laboratory characteristics of patients with type 1 diabetes mellitus (T1DM) without and with diabetic retinopathy (DR)

Characteristics	Without DR (controls; n = 129)	With DR (cases; n = 107)	p*
Age (years)	34.6 ± 12.2	41.4 ± 12.1	0.0001
Gender (% male)	53.5	52.3	0.964
BMI (kg/m²)	24.3 ± 3.5	24.0 ± 3.5	0.656
HbA1c (%)	8.4 ± 1.6	8.8 ± 1.9	0.037
Hypertension (%)	39.4	58.7	0.005
T1DM duration (years)	19.4 ± 9.1	23.5 ± 7.6	0.0001
Total cholesterol (mmol/L)	4.3 ± 1.0	4.9 ± 1.3	0.0001
Triglycerides (mmol/L)	0.7 (0.6-1.0)	1.1 (0.7-1.5)	0.0001
HDL cholesterol (mmol/L)	1.4 ± 0.4	1.5 ± 0.5	0.139
LDL cholesterol (mmol/L)	2.5 ± 0.7	2.8 ± 1.0	0.011
eGFR (mL/min per 1.73 m^2^)	103.0 (86.0-117.0)	83.0 (56.5-109.0)	0.0001
UAE (mg/mmol)	0.8 (0.5-2.1)	1.7 (0.6-22.0)	0.0001

Variables are shown as means ± standard deviations (SDs), medians (25th-75th percentiles), or %.

* p-values were computed using Student’s *t* tests or χ^2^ tests, as appropriate. BMI: body mass index; eGFR: estimated glomerular filtration rate; HbA1c: glycated hemoglobin; HDL: high-density lipoprotein; LDL: low-density lipoprotein; UAE: urinary albumin excretion.

### Genotype and allele frequencies

Table 2 describes genotype and allele frequencies of the rs2442598 SNP in the *ANGPT-2* gene in cases and controls. Genotype distributions of this SNP were in agreement with those predicted by the HWE in both groups (p ≥ 0.05). The frequency of the A/A genotype was higher in cases than in controls (10.3% vs. 2.3%, p = 0.017; Table 2), and it was associated with risk for DR under the recessive (p = 0.022) and additive (p = 0.013) genetic models. Additionally, the frequency of the A allele was also increased in patients with DR compared to controls (30% vs. 20%, p = 0.011; Table 1).

**Table 2:** Genotype and allele frequencies of the *ANGPT-2* rs2442598 single nucleotide polymorphism (SNP) in patients with type 1 diabetes mellitus (T1DM) without and with diabetic retinopathy (DR)

	T1DM		Unadjusted p*	Adjusted OR (95% CI)/p†
	Without DR (controls) (n = 129)	With DR (cases) (n = 107)		
**Genotype**				
G/G	80 (62.0)	53 (49.5)	0.017	1
G/A	46 (35.7)	43 (40.2)		1.222 (0.640-2.336)/0.544
A/A	3 (2.3)	11 (10.3)		5.190 (1.210-22.269)/0.027
**Allele**				
G	0.80	0.70	0.011	
A	0.20	0.30		

Data are shown as numbers (%) or proportions.

* p-values were calculated using χ^2^ tests.

† p-values and odds ratios (OR) (95% confidence interval [CI]) obtained using logistic regression analyses adjusting for duration of T1DM, glycated hemoglobin (HbA1c), triglycerides, estimated glomerular filtration rate (eGFR), and presence of hypertension.

**Figure 1:**
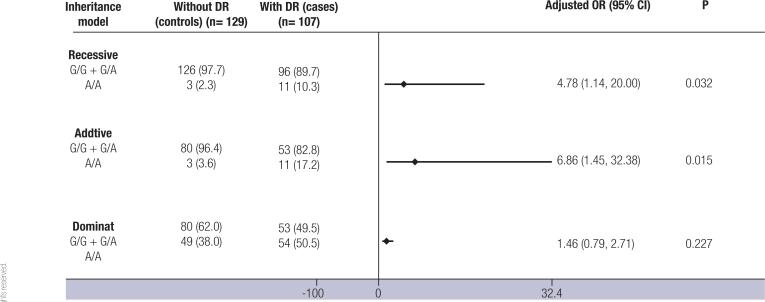
Frequencies of the *ANGPT-2* rs2442598 single nucleotide polymorphism (SNP) in patients with type 1 diabetes mellitus (T1DM) without (controls) or with diabetic retinopathy (DR) (cases), categorized according to different inheritance models. Data are shown as numbers (%) or proportions. p-values and odds ratios (OR, 95% confidence interval [CI]) were obtained using logistic regression analyses adjusting for duration of T1DM, glycated hemoglobin (HbA1c), triglycerides, estimated glomerular filtration rate (eGFR), and presence of hypertension.

Moreover, after adjustment for the duration of DM, HbA1c, triglycerides, eGFR, and presence of hypertension, the A/A genotype remained associated with risk for DR (OR = 5.190, 95% confidence interval [CI] 1.210-22.269; p = 0.027; Table 1). Regarding the genetic inheritance models, this SNP was also associated with DR under recessive (OR = 4.779, 95% CI 1.142–19.998; p = 0.032) and additive (OR = 6.861, 95% CI 1.454-32.378; p = 0.015) models after adjustment for the aforementioned variables (Figure 1). However, the rs2442598 SNP was not associated with DR under the dominant model (p = 0.073), and this result did not change after adjusting for covariables (OR = 1.464, 95% CI 0.789-2.715; p = 0.227; Figure 1).

In an exploratory analysis, the clinical and laboratory characteristics of patients with T1DM, regardless of their DR status, were compared after stratification according to the presence of the rs2442598 A/A genotype under the recessive model (Table 3). The frequency of male participants and mean values of body mass index (BMI), HbA1c, age at T1DM diagnosis, duration of DM, total cholesterol, triglycerides, HDL cholesterol, LDL cholesterol, eGFR, and UAE were similar between patients with A/A or G/G + G/A genotypes (p > 0.050). Interestingly, the presence of hypertension was more frequent in patients carrying the A/A genotype in comparison with patients carrying the G/G + G/A genotypes (76.9% vs. 44.3%, p = 0.045).

**Table 3:** Clinical and laboratory characteristics of patients with type 1 diabetes mellitus (T1DM) stratified by the presence of the A allele of the *ANGPT-2* rs2442598 single nucleotide polymorphism (SNP) (recessive model)

Characteristics	G/G + G/A (n = 250)	A/A (n = 16)	p*
Age (years)	37.8 ± 12.5	38.8 ± 12.2	0.782
Gender (% male)	52.9	57.1	0.974
BMI (kg/m²)	24.1 ± 3.4	24.7 ± 4.2	0.594
HbA1c (%)	8.5 ± 1.8	8.3 ± 2.4	0.593
Hypertension (%)	44.3	76.9	0.045
T1DM duration (years)	20.9 ± 8.8	21.9 ± 7.0	0.724
Total cholesterol (mmol/L)	4.6 ± 1.2	4.5 ± 0.8	0.709
Triglycerides (mmol/L)	0.9 (0.6-1.4)	0.7 (0.6-1.6)	0.635
HDL cholesterol (mmol/L)	1.4 ± 0.4	1.6 ± 0.4	0.156
LDL cholesterol (mmol/L)	2.6 ± 0.9	2.5 ± 0.7	0.842
eGFR (mL/min per 1.73 m^2^)	94.0 (73.0-113.5)	95.0 (72.5-121.5)	0.410
UAE (mg/mmol)	1.1 (0.6-5.0)	5.5 (0.8-10.3)	0.454

Variables are shown as means ± SDs, medians (25th-75th percentiles), or %.

* p-values were computed using Student’s *t* tests or χ^2^ tests, as appropriate. BMI: body mass index; HDL: high-density lipoprotein; LDL: low-density lipoprotein; eGFR: estimated glomerular filtration rate; HbA1c: glycated hemoglobin; UAE: urinary albumin excretion.

## DISCUSSION

Our results demonstrated, for the first time, that the A allele of the rs2442598 SNP in the *ANGPT-2* gene is associated with risk for DR in patients with T1DM from southern Brazil. Although the exact role of *ANGPT-2* in angiogenesis and its involvement in disease mechanisms are not fully understood, there is enough data to link ANGPT-2 to DM and its vascular complications (10,24-26).

El-Asrar and cols. (24) evaluated serum ANGPT-2 in children and adolescents with T1DM as a potential marker of vascular complications. Compared with healthy controls, ANGPT-2 levels were higher in patients with T1DM and were positively correlated with fasting blood glucose and HbA1c levels. When comparing patients with T1DM with or without vascular complications, the highest ANGPT-2 levels were observed in those with complications. ANGPT-2 also had a positive correlation with carotid and aortic intima-media thickness (24). In T2DM patients, a study showed that serum ANGPT-2 levels were higher in patients with cardiovascular disease (CVD) compared to those without CVD or healthy controls. Moreover, ANGPT-2 levels were associated with a 2.83-fold higher risk of T2DM and were positively correlated with fasting blood glucose, HbA1c, homeostatic model assessment of insulin resistance (HOMA-IR), triglycerides, and LDL cholesterol (25).

The involvement of ANGPT-2 in PDR has also been well demonstrated. Klaassen and cols. (26) studied vitreous samples from patients with PDR (with and without fibrovascular membrane [FVM] formation) and from non-diabetic patients with vitreous debris to identify proteins associated with neurite outgrowth, angiogenesis, and fibrosis in the formation of FVM in PDR. Among 8 other associated proteins, ANGPT-2 showed the strongest correlation with the degree of fibrosis and presence of FVM in patients with PDR, pointing to an additional role in the development of PDR apart from active angiogenesis (26). Another study demonstrated increased ANGPT-2 levels in patients with DM compared to healthy controls, with the highest ANGPT-2 levels being observed among patients with pre-proliferative or PDR (27). In contrast, Malik and cols. (28) did not find any differences in serum ANGPT-2 levels between patients with diabetes with or without DR.

Another evidence of the involvement of ANGPT-2 in DR is related to its effect on pericyte loss in the early phases of DR development. An experimental study conducted by Hammes and cols. (10) demonstrated that Angpt-2 was upregulated more than 30-fold in the retina of diabetic rats when compared to nondiabetic controls and was associated with pericyte loss in DR. When recombinant Angpt-2 was injected into the eyes of normal rats, pericyte loss was observed in a dose-dependent manner (10). Park and cols. (29), studying the molecular mechanisms behind the role of hyperglycemia-induced Angpt-2 in pericyte loss, demonstrated that it could induce pericyte apoptosis via the p53 pathway.

Furthermore, Angpt-2 was associated with astrocyte loss in DR. Astrocytes are neurovascular components of the retina that contribute to vessel integrity and are closely related to the formation of tight junctions in the retina (30). Yun and cols. (30) studied the role of ANGPT-2 in astrocyte loss and vascular leakage in the retina of early diabetic mice. They were able to show that, as Angpt-2 increased, vascular leakage with loss of astrocytes occurred and was induced by apoptosis via the GSK-3β/β-catenin pathway under high glucose conditions. Similarly, Rangasamy and cols. (9) demonstrated the ability of Angpt-2 to alter the retinal endothelial cell barrier *in vitro* and *in vivo*. Retinal endothelial cells cultured with increasing concentrations of purified recombinant ANGPT-2 showed a dose-dependent increase in monolayer permeability. Moreover, a threefold increase in vascular permeability was observed when nondiabetic rats received a single intraocular injection of recombinant purified Angpt-2 (9). Hence, ANGPT-2 seems to be closely involved with the damage and loss of pericytes and astrocytes that directly participate in the pathogenesis of DR.

This study has a few limitations. First, we cannot rule out the possibility of population stratification bias when analyzing our samples, even though only White participants were studied and both the cases and controls were recruited from the same hospital, thus reducing the risk of false-positive/negative associations due to this bias. Second, this is the first study that demonstrated an association between the rs2442598 SNP in the *ANGPT-2* gene and risk for DR, and we did not perform a replication of the observed association in another Brazilian sample. Third, there is a lack of information about how rs2442598 affects *ANGPT-2* expression and what is its functional role on DR susceptibility. Therefore, additional genetic studies are needed in order to confirm the association between the rs2442598 SNP in *ANGPT-2* and the risk for DR in different ethnicities and populations. Moreover, functional studies are necessary to better understand how rs2442598 affects ANGPT-2 expression and activity.

In conclusion, the present data suggest an association between the A allele of the rs2442598 SNP in *ANGPT-2* and susceptibility to DR in patients with T1DM. To our knowledge, this is the first report of a polymorphism study of the *ANGPT-2* gene suggesting risk for DR. This finding needs to be further evaluated in other populations, although there is a good rationale for this association to be relevant given the role of ANGPT-2 in the mechanisms involved in DR pathogenesis.

## References

[1] Cheung N, Mitchell P, Wong TY (2010). Diabetic retinopathy. Lancet.

[2] Saeedi P, Petersohn I, Salpea P, Malanda B, Karuranga S, Unwin N (2019). Global and regional diabetes prevalence estimates for 2019 and projections for 2030 and 2045: Results from the International Diabetes Federation Diabetes Atlas, 9(th) edition. Diabetes Res Clin Pract.

[3] Priscakova P, Minarik G, Repiska V (2016). Candidate gene studies of diabetic retinopathy in human. Mol Biol Rep.

[4] Huang H, Bhat A, Woodnutt G, Lappe R (2010). Targeting the ANGPT-TIE2 pathway in malignancy. Nat Rev Cancer.

[5] Whitehead M, Osborne A, Widdowson PS, Yu-Wai-Man P, Martin KR (2019). Angiopoietins in Diabetic Retinopathy: Current Understanding and Therapeutic Potential. J Diabetes Res.

[6] Thurston G, Daly C (2012). The complex role of angiopoietin-2 in the angiopoietin-tie signaling pathway. Cold Spring Harb Perspect Med.

[7] Isidori AM, Venneri MA, Fiore D (2016). Angiopoietin-1 and Angiopoietin-2 in metabolic disorders: therapeutic strategies to restore the highs and lows of angiogenesis in diabetes. J Endocrinol Invest.

[8] Maisonpierre PC, Suri C, Jones PF, Bartunkova S, Wiegand SJ, Radziejewski C (1997). Angiopoietin-2, a natural antagonist for Tie2 that disrupts in vivo angiogenesis. Science.

[9] Rangasamy S, Srinivasan R, Maestas J, McGuire PG, Das A (2011). A potential role for angiopoietin 2 in the regulation of the blood-retinal barrier in diabetic retinopathy. Invest Ophthalmol Vis Sci.

[10] Hammes HP, Lin J, Wagner P, Feng Y, Vom Hagen F, Krzizok T (2004). Angiopoietin-2 causes pericyte dropout in the normal retina: evidence for involvement in diabetic retinopathy. Diabetes.

[11] Dai C, Kuo SJ, Zhao J, Jin L, Kang L, Wang L (2019). Correlation between genetic polymorphism of angiopoietin-2 gene and clinical aspects of rheumatoid arthritis. Int J Med Sci.

[12] He L, Dang L, Zhou J, Bai J, Li YZ (2015). Association of angiopoietin-1, angiopoietin-2 and caspase-5 polymorphisms with psoriasis vulgaris. Clin Exp Dermatol.

[13] Meyer NJ, Li M, Feng R, Bradfield J, Gallop R, Bellamy S (2011). ANGPT2 genetic variant is associated with trauma-associated acute lung injury and altered plasma angiopoietin-2 isoform ratio. Am J Respir Crit Care Med.

[14] Little J, Higgins JP, Ioannidis JP, Moher D, Gagnon F, von Elm E (2009). STrengthening the REporting of Genetic Association Studies (STREGA) – an extension of the STROBE statement. Genet Epidemiol.

[15] von Elm E, Altman DG, Egger M, Pocock SJ, Gotzsche PC, Vandenbroucke JP (2008). The Strengthening the Reporting of Observational Studies in Epidemiology (STROBE) statement: guidelines for reporting observational studies. J Clin Epidemiol.

[16] American Diabetes Association (2019). 2. Classification and Diagnosis of Diabetes: Standards of Medical Care in Diabetes-2019. Diabetes Care.

[17] Wilkinson CP, Ferris FL, Klein RE, Lee PP, Agardh CD, Davis M (2003). Proposed international clinical diabetic retinopathy and diabetic macular edema disease severity scales. Ophthalmology.

[18] Boucas AP, Brondani LA, Souza BM, Lemos NE, de Oliveira FS, Canani LH (2013). The A allele of the rs1990760 polymorphism in the IFIH1 gene is associated with protection for arterial hypertension in type 1 diabetic patients and with expression of this gene in human mononuclear cells. PLoS One.

[19] Camargo JL, Zelmanovitz T, Paggi A, Friedman R, Gross JL (1998). Accuracy of conversion formulae for estimation of glycohaemoglobin. Scand J Clin Lab Invest.

[20] Zelmanovitz T, Gross JL, Oliveira JR, Paggi A, Tatsch M, Azevedo MJ (1997). The receiver operating characteristics curve in the evaluation of a random urine specimen as a screening test for diabetic nephropathy. Diabetes Care.

[21] Levey AS, Stevens LA, Schmid CH, Zhang YL, Castro AF, Feldman HI (2009). A new equation to estimate glomerular filtration rate. Ann Intern Med.

[22] Lahiri DK, Nurnberger JI (1991). A rapid non-enzymatic method for the preparation of HMW DNA from blood for RFLP studies. Nucleic Acids Res.

[23] Zintzaras E, Lau J (2008). Synthesis of genetic association studies for pertinent gene-disease associations requires appropriate methodological and statistical approaches. J Clin Epidemiol.

[24] El-Asrar MA, Elbarbary NS, Ismail EA, Bakr AA (2016). Circulating angiopoietin-2 levels in children and adolescents with type 1 diabetes mellitus: relation to carotid and aortic intima-media thickness. Angiogenesis.

[25] El-Lebedy D (2019). Association of serum angiopoietin-like protein 2 with elevated risk of cardiovascular diseases in subjects with type 2 diabetes. J Diabetes Complications.

[26] Klaassen I, de Vries EW, Vogels IMC, van Kampen AHC, Bosscha MI, Steel DHW (2017). Identification of proteins associated with clinical and pathological features of proliferative diabetic retinopathy in vitreous and fibrovascular membranes. PLoS One.

[27] Lip PL, Chatterjee S, Caine GJ, Hope-Ross M, Gibson J, Blann AD (2004). Plasma vascular endothelial growth factor, angiopoietin-2, and soluble angiopoietin receptor tie-2 in diabetic retinopathy: effects of laser photocoagulation and angiotensin receptor blockade. Br J Ophthalmol.

[28] Malik TG, Ahmed SS, Gul R, Ayesha E (2018). Comparative Analysis of Serum Proangiogenic Biomarkers between those with and without Diabetic Retinopathy. J Coll Physicians Surg Pak.

[29] Park SW, Yun JH, Kim JH, Kim KW, Cho CH, Kim JH (2014). Angiopoietin 2 induces pericyte apoptosis via alpha3beta1 integrin signaling in diabetic retinopathy. Diabetes.

[30] Yun JH, Park SW, Kim JH, Park YJ, Cho CH, Kim JH (2016). Angiopoietin 2 induces astrocyte apoptosis via alphavbeta5-integrin signaling in diabetic retinopathy. Cell Death Dis.

